# Moderating Role of Job Satisfaction on Organizational Climate and Professional Identity in Nurses: A Multicenter Cross‐Sectional Study

**DOI:** 10.1155/jonm/5273090

**Published:** 2026-06-26

**Authors:** Chong Chen, Huijun Liu, Juan Lai

**Affiliations:** ^1^ Teaching and Research Section of Clinical Nursing, Xiangya Hospital of Central South University, Changsha, 410008, Hunan, China, csu.edu.cn

**Keywords:** job satisfaction, moderating effect, nursing management, organizational climate, professional identity

## Abstract

**Background:**

Organizational climate plays a key role in shaping nurses’ professional identity, which is essential in mitigating postpandemic nursing shortages. However, whether job satisfaction moderates the association between organizational climate and professional identity remains underexplored in Chinese tertiary hospitals.

**Aim:**

To explore the association between organizational climate and nurses’ professional identity and to verify the moderating effect of job satisfaction on this relationship, grounded in the Job Demands–Resources framework and the Person–Environment Fit Theory.

**Methods:**

This multicenter cross‐sectional study recruited 580 nurses across five tertiary hospitals in Changsha, Hunan Province, between July and August 2025. Organizational climate, professional identity, and job satisfaction were assessed using the Nurse Organizational Climate Scale, the Nurse Professional Identity Rating Scale, and the Minnesota Satisfaction Questionnaire (Short‐Form), respectively. The moderation model was tested using Spearman’s rank correlation and hierarchical linear regression.

**Results:**

Nurses exhibited a moderate level of professional identity, with a median score of 109.0 (IQR: 90.0–120.0). Organizational climate was positively correlated with professional identity (*r* = 0.681, *p* < 0.001), and such an association was positively moderated by job satisfaction (*β* = 0.086, *p* < 0.01). Specifically, the positive association between organizational climate and professional identity became stronger with increasing levels of job satisfaction.

**Conclusion:**

The organizational climate is positively associated with professional identity, and job satisfaction moderates this relationship. Our findings carry significant implications for future interventions to improve nurses’ professional identity, suggesting a dual‐strategy approach that optimizes the objective organizational climate while simultaneously enhancing nurses’ subjective job satisfaction.

**Implications for Nursing Management:**

Foster a supportive organizational climate (e.g., resource guarantee, shared decision‐making, and transformational leadership) and systematically enhance job satisfaction (e.g., emotional support, professional supervision, and fair incentives). A dual‐strategy approach targeting both the environment and subjective satisfaction is key to strengthening nurses’ professional identity and addressing workforce shortages.

## 1. Introduction

The global nursing shortage is a significant public health challenge, exacerbated by the post‐COVID‐19 pandemic strain and increasing healthcare demands [1]. According to the World Health Organization (WHO), the global nursing shortage reached 5.8 million in 2023, disproportionately affecting low‐ and middle‐income countries (LMICs) [2]. The International Council of Nurses (ICN) projects a need for 13 million additional nurses over the next 10 years, declaring the current nursing shortage a “global health emergency.”

A sustainable solution to the nursing shortage requires a transition from merely expanding recruitment to cultivating professional identity [3]. Despite the well‐established link between organizational climate and professional identity, few empirical studies have examined whether job satisfaction moderates their association, particularly in the highly demanding environment of tertiary hospitals in China. Understanding these dynamics is essential for developing evidence‐based strategies that improve both environmental and psychological factors to strengthen the nursing workforce [4]. Therefore, this study aims to examine the association between organizational climate and professional identity, with a focus on the moderating effect of job satisfaction.

## 2. Background

### 2.1. Professional Identity in Nursing

Professional identity in nursing is defined as the internalization of the profession’s core domains: values and ethics, knowledge, leadership, and comportment [5]. As a key driver of workforce stability, it represents a developmental shift from merely performing clinical tasks to fully embodying the role of a nurse [5]. Nurses with higher levels of professional identity not only have higher psychological resilience and job satisfaction but also lower job burnout and turnover rates, which ultimately improve clinical performance and patient outcomes [6]. Therefore, exploring the factors that influence professional identity is imperative for addressing the current global health emergency.

### 2.2. Organizational Climate and the Job Demands–Resources (JD‐R) Framework

Organizational climate is a well‐established factor that influences the formation of professional identity [7]. Organizational climate refers to the collective perceptions and shared understandings of staff members regarding their workplace’s policies, practices, and procedures [8]. In healthcare, a supportive organizational climate enables nurses to internalize professional values and strengthen their professional identity [7]. This beneficial role is grounded in the JD‐R framework, wherein organizational support serves as a critical job resource that reduces psychological stress and fosters personal growth [9]. A supportive environment motivates work engagement and facilitates the internalization of professional values [10]. This resource‐driven motivation is essential for the long‐term maintenance of professional identity, particularly in highly demanding clinical settings where emotional distress is prevalent [11].

Based on this evidence, we propose the following:

**Hypothesis 1.**

Organizational climate is positively associated with nurses’ professional identity.

### 2.3. The Moderating Role of Job Satisfaction

Despite the established link between organizational climate and professional identity, the specific mechanism underlying their association remains complex and multifaceted. The Person–Environment fit theory suggests that an individual’s subjective experience may affect the environment’s impact [12]. Consequently, the association between organizational climate and professional identity may vary depending on a nurse’s internal perception. Job satisfaction—defined as the degree to which individuals feel satisfied in their work roles—may act as a critical moderator that regulates the internalization of professional values. Specifically, high levels of satisfaction strengthen the positive impact of a supportive organizational climate on professional identity, while low satisfaction may alleviate these benefits [13]. Understanding this interaction is essential to guiding effective and targeted organizational interventions to improve professional identity.

Based on this evidence, we propose the following:

**Hypothesis 2.**

Job satisfaction positively moderates the association between organizational climate and professional identity.

## 3. Methods

### 3.1. Study Design and Participants

A cross‐sectional study was conducted in tertiary general hospitals in Changsha, Hunan Province, China, between July and August 2025. To enhance the generalizability of the findings, a multistage stratified random sampling method was utilized. In the first stage, five representative tertiary hospitals were selected as the primary sampling units. In the second stage, departments were stratified into nine clusters (internal medicine, surgery, emergency, ICU, pediatrics, operating room, ENT/ophthalmology, outpatient, and obstetrics and gynecology). Finally, within each cluster, participants were randomly selected using a computer‐generated random number table. This rigorous approach minimizes selection bias and ensures the sample representativeness.

Participants were included if they satisfied the following inclusion criteria: (1) registered nurses with a valid professional nursing license; (2) ≥ six months of clinical nursing experience; and (3) voluntary participation in the study. The exclusion criteria were as follows: (1) nurses on extended leave (e.g., sick leave and maternity leave) for more than 3 months during the survey period; (2) visiting nurses, nursing students, and trainees undergoing standardized residency training; and (3) nurses who had experienced major adverse life events (e.g., divorce, serious illness, or bereavement involving a spouse, child, or parent) within the past 3 months. The sample size was determined using Kendall’s criteria for multivariate research, which stipulate that the sample should be at least 10–15 times the number of independent variables to ensure adequate statistical power and stable parameter estimates. Given that our model included 17 variables, we adopted the more conservative 15:1 ratio. After adjusting for a projected 30% nonresponse rate and an anticipated 80% questionnaire validity rate, the final target sample was set at 455 participants.

### 3.2. Data Collection Procedure

This study was approved by the Ethics Committee of Xiangya Hospital, Central South University (Approval No. 202312254). All participants provided informed consent and participated voluntarily. Before data collection, authorization was obtained from the nursing department directors of the participating hospitals. Three uniformly trained researchers were deployed to the hospitals to coordinate with unit head nurses. The head nurses received training on the survey protocol, including the distribution method, inclusion/exclusion criteria, and the need to emphasize anonymity, confidentiality, and independent completion to the staff.

The survey was administered electronically using SoJump, one of the biggest online survey platforms in China. The questionnaire included clear instructions on the introductory page and at the beginning of each section. To ensure broad accessibility and real‐time dissemination across nursing shifts, the digital survey was distributed via WeChat (Tencent; Shenzhen, China), the predominant mobile communication platform in the Chinese healthcare sector. The head nurses of each clinical unit facilitated the distribution of the survey link within dedicated professional departmental groups. This administrative‐led approach has been identified as an effective strategy for maximizing response rates and ensuring data collection consistency within large‐scale tertiary hospital systems [14].

To ensure data quality, the platform was configured to allow only one submission per IP address and to restrict submissions until all items were completed. The data collection window for each unit was limited to 72 h. Upon completion of the survey, the three researchers conducted a rigorous manual review of the collected data. Questionnaires were excluded if they showed suspicious response patterns (e.g., straight‐lining or identical answers to all items) or if the completion time was less than 300 s. A total of 600 questionnaires were distributed. After quality screening, 580 valid questionnaires were retained, yielding an effective response rate of 96.7%.

### 3.3. Measurement Instruments

#### 3.3.1. General Demographic Questionnaire

A self‐designed demographic form was utilized to collect participants’ demographic and work‐related characteristics. Demographic characteristics included age, educational level, marital status, and number of children. Work‐related variables included years of nursing experience, professional title, clinical department, weekly working hours, daily patient volume, daily patient contact duration, part‐time status, annual shift type, monthly night‐shift frequency, and monthly income.

#### 3.3.2. Nurse Organizational Climate Scale (NOCS)

The perceived organizational environment was assessed using the NOCS developed by He et al. [15]. It includes 37 items under six dimensions: resource guarantee (10 items), team behavior (8 items), management support (9 items), quality management (4 items), human resource management (4 items), and evidence‐based nursing support (2 items). Responses are rated on a four‐point Likert scale ranging from 1 (“*strongly disagree*”) to 4 (“*strongly agree*”). Total scores are calculated by summing the item scores, with higher values reflecting a more favorable perception of the organizational climate. The scale demonstrated robust psychometric properties, with an overall Cronbach’s alpha of 0.939, subdimensional alpha coefficients ranging from 0.745 to 0.877, test–retest reliability of 0.813, and split‐half reliability of 0.902.

#### 3.3.3. Nurse Professional Identity Scale (NPIS)

Nurse professional identity was measured using the NPIS developed by Liu et al. [16]. It includes 30 items under five domains: professional self‐concept (9 items), professional social support (6 items), professional social skills (6 items), professional frustration coping (6 items), and professional self‐reflection (3 items). A 5‐point Likert scale is employed, ranging from 1 (“*strongly disagree/does not match at all*”) to 5 (“*strongly agree/matches perfectly*”), with total scores ranging from 30 to 150. Based on these scores, professional identity is categorized into four levels: low (30–60), relatively low (61–90), moderate (91–120), and high (121–150). Higher scores indicate a stronger sense of professional identity. The scale’s reliability was confirmed by an overall Cronbach’s alpha of 0.938, subdimensional alpha coefficients > 0.70, and split‐half reliability > 0.88.

#### 3.3.4. Minnesota Satisfaction Questionnaire Short Form (MSQ‐SF)

Job satisfaction was evaluated using the MSQ‐SF, initially developed by Weiss et al. [17] and translated into Chinese by Zhou et al. [18]. The 20‐item instrument encompasses three dimensions: intrinsic satisfaction (12 items), extrinsic satisfaction (6 items), and general satisfaction (2 items). Items are rated on a five‐point Likert scale from 1 (“*very dissatisfied*”) to 5 (“*very satisfied*”). The total score ranges from 20 to 100, with higher scores indicating greater job satisfaction. In the present study, the instrument demonstrated excellent internal consistency (Cronbach’s alpha = 0.959).

### 3.4. Statistical Analysis

Data were entered using a double‐entry verification method to ensure accuracy and were analyzed using SPSS, Version 25.0 (IBM Corp., Armonk, NY, USA). Categorical variables were expressed as frequencies and percentages. All continuous variables were reported as medians and interquartile ranges (IQRs) because they were non‐normal. To compare differences in professional identity scores across demographic groups, the Mann–Whitney *U* test (for two groups) or the Kruskal–Wallis H test (for three or more groups) was employed. Spearman’s rank correlation coefficient was used to examine the relationships between organizational climate, job satisfaction, and professional identity.

Hierarchical linear regression analysis was employed to examine the moderating effect of job satisfaction on the relationship between organizational climate and professional identity. Before the analysis, the independent variable (organizational climate) and the moderator (job satisfaction) were mean‐centered, and an interaction term was calculated as their product. The regression was conducted in three steps. Model 1 included significant demographic and occupational variables as control variables. Model 2 added organizational climate and job satisfaction to the equation. Model 3 introduced the interaction term (organizational climate ^∗^ job satisfaction) to assess the moderating effect. All tests were two‐tailed, with alpha = 0.05. A *p* value < 0.05 was considered statistically significant. Multicollinearity was assessed using the variance inflation factor (VIF) and tolerance. A VIF value below 5 and a tolerance above 0.2 were considered acceptable, indicating no serious multicollinearity. Because all continuous variables were standardized to Z scores before analysis, the unstandardized coefficients (B) are equal to the standardized coefficients (*β*).

## 4. Results

### 4.1. Normality Assessment and Comparison of Professional Identity Scores by Demographics

Before conducting group comparisons, we examined the distribution of the three key continuous variables. As shown in Table 1, all variables significantly deviated from a normal distribution. The absolute values of skewness‐to‐standard‐error and kurtosis‐to‐standard‐error ratios exceeded 1.96 for the key variables, indicating significant departures from normality. This was further supported by the Shapiro–Wilk and Kolmogorov–Smirnov tests. Consequently, nonparametric tests (Mann–Whitney U, Kruskal–Wallis H, and Spearman’s rank correlation) were used for subsequent analyses.

**TABLE 1:** Normality tests for key study variables (*N* = 580).

Key variables	Standard errors (skewness/kurtosis)	Skewness	Kurtosis	*S–W*	*K–S*	*p*
Organizational climate	SE_s_ = 0.101/SE_k_ = 0.203	−0.584	0.880	0.935	0.123	< 0.001
Job satisfaction	SE_s_ = 0.101/SE_k_ = 0.203	−0.317	1.518	0.964	0.101	< 0.001
Professional identity	SE_s_ = 0.101/SE_k_ = 0.203	−0.565	1.102	0.939	0.108	< 0.001

*Note:* The Shapiro–Wilk (S–W) test was the primary test for normality, with the Kolmogorov–Smirnov (K–S) test used as a supplementary test. A *p* value < 0.05 indicates a significant deviation from the normal distribution. *p* values refer to both Shapiro–Wilk and Kolmogorov–Smirnov tests if both were significant.

Abbreviations: SEk, standard error of kurtosis; SEs, standard error of skewness.

Nurses exhibited a moderate level of professional identity, with a median total score of 109.0 (IQR: 90.0, 120.0). When examining the five dimensions of the NPIS, the median scores were as follows: professional self‐concept at 33.0 (IQR: 27.0, 36.0), professional social skills at 22.0 (IQR: 18.0, 24.0), professional frustration coping at 22.0 (IQR: 18.0, 24.0), professional social support at 21.0 (IQR: 18.0, 24.0), and professional self‐reflection at 11.0 (IQR: 9.0, 12.0). Comparison of professional identity scores across different demographic and occupational groups showed significant differences by annual shift type (H = 14.949, *p* = 0.001) and monthly night‐shift frequency (H = 13.899, *p* = 0.008). Specifically, nurses working routine day shifts and those with no night shifts had significantly higher professional identity scores than their counterparts. Detailed comparisons are presented in Table 2.

**TABLE 2:** Comparison of nurse professional identity scores across different demographic characteristics (*N* = 580).

Variable	*n* (%)	M (P25, P75)	H/Z	*p*
Age (years)			7.959	0.093
20–25	74 (12.8)	110.5 (90.0, 120.0)		
26–30	241 (41.6)	109.0 (90.0, 120.0)		
31–35	140 (24.1)	102.5 (90.0, 120.0)		
36–40	67 (11.6)	110.0 (90.0, 120.0)		
> 40	58 (10.0)	115.0 (96.8, 133.0)		
Education level			3.962	0.266
Secondary school	6 (1.0)	106.5 (96.8, 126.3)		
Junior college	88 (15.2)	115.0 (93.0, 120.8)		
Bachelor’s degree	451 (77.8)	108.0 (90.0, 120.0)		
Master’s and above	35 (6.0)	113.0 (90.0, 131.0)		
Marital status			2.703	0.259
Unmarried	151 (26.0)	109.0 (90.0, 120.0)		
Married	415 (71.6)	110.0 (90.0, 120.0)		
Divorced	14 (2.4)	97.0 (90.0, 110.0)		
Number of children			1.303	0.728
0	207 (35.7)	113.0 (90.0, 120.0)		
1	266 (45.9)	106.5 (90.0, 120.0)		
2	105 (18.1)	108.0 (90.0, 120.0)		
> 2	2 (0.3)	94.5 (69.0, 120.0)		
Department			7.366	0.498
Outpatient	72 (12.4)	110.0 (90.0, 120.8)		
Obstetrics and gynecology	62 (10.7)	106.5 (90.0, 120.0)		
Internal medicine	72 (12.4)	109.5 (90.0, 120.0)		
Emergency	44 (7.6)	112.5 (92.3, 120.0)		
Surgery	121 (20.9)	102.0 (90.0, 120.0)		
Pediatrics	24 (4.1)	106.5 (96.0, 121.5)		
ICU	80 (13.8)	105.5 (90.0, 120.0)		
Operating room	71 (12.2)	115.0 (94.0, 124.0)		
ENT/ophthalmology	34 (5.9)	113.5 (95.5, 122.5)		
Professional title			0.687	0.876
Nurse	78 (13.4)	114.0 (90.0, 120.3)		
Senior nurse	285 (49.1)	109.0 (90.0, 120.0)		
Supervisor nurse	214 (36.9)	108.0 (90.0, 120.0)		
Associate chief nurse	3 (0.5)	109.0 (90.0, 139.0)		
Years of experience			4.74	0.315
< 1	6 (1.0)	120.0 (89.5, 125.3)		
1–5	138 (23.8)	112.5 (90.0, 120.0)		
6–10	249 (42.9)	104.0 (90.0, 120.0)		
11–15	90 (15.5)	108.0 (90.0, 120.3)		
> 15	97 (16.7)	112.0 (95.5, 120.0)		
Work hours/week			5.673	0.225
< 30	9 (1.6)	120.0 (100.5, 123.0)		
31–35	188 (32.4)	110.5 (92.0, 120.0)		
36–40	290 (50.0)	109.0 (90.0, 120.0)		
41–45	87 (15.0)	105.0 (90.0, 120.0)		
> 50	6 (1.0)	92.5 (75.8, 100.8)		
Patients/day (*n*)			3.851	0.278
1–4	158 (27.2)	112.0 (90.0, 120.0)		
5–10	154 (26.6)	111.0 (90.0, 120.0)		
11–15	119 (20.5)	99.0 (90.0, 120.0)		
> 15	149 (25.7)	109.0 (90.0, 120.0)		
Contact time/day (h)			2.634	0.452
1–5	39 (6.7)	114.0 (96.0, 120.0)		
6–10	504 (86.9)	108.0 (90.0, 120.0)		
11–15	19 (3.3)	98.0 (90.0, 125.0)		
> 15	18 (3.1)	100.0 (93.3, 120.0)		
Annual shift type			14.949	0.001^∗∗^
Rotating shift	397 (68.4)	104.0 (90.0, 120.0)		
Routine day shift	167 (28.8)	116.0 (96.0, 124.0)		
On leave	16 (2.8)	106.0 (87.8, 120.0)		
Night shifts/month (*n*)			13.899	0.008^∗∗^
0	168 (29.0)	114.0 (93.3, 122.8)		
1‐2	104 (17.9)	111.5 (92.0, 120.0)		
3–5	205 (35.3)	101.0 (90.0, 120.0)		
6–10	75 (12.9)	110.0 (90.0, 120.0)		
> 10	28 (4.8)	110.0 (90.0, 120.0)		
Monthly income (RMB)			2.092	0.719
< 5000	40 (6.9)	110.0 (94.0, 121.8)		
5000–10,000	413 (71.2)	109.0 (90.0, 120.0)		
10,001–15,000	105 (18.1)	109.0 (90.0, 120.0)		
15,001–20,000	19 (3.3)	112.0 (98.0, 120.0)		
> 20,000	3 (0.5)	103.0 (76.0, 110.0)		
Part‐time status			−1.045	0.296
Yes	17 (2.9)	117.0 (94.0, 125.0)		
No	563 (97.1)	109.0 (90.0, 120.0)		

*Note:* M = median; P_25, P_75 = 25th and 75th percentiles (interquartile range).

^∗∗^ *p* < 0.01.

### 4.2. Descriptive Statistics and Correlation Analysis of Study Variables

As shown in Table 3, the median scores for organizational climate, professional identity, and job satisfaction were 110.0 (IQR: 97.0, 118.0), 109.0 (IQR: 90.0, 120.0), and 76.0 (IQR: 68.0, 80.0), respectively. Spearman’s rank correlation analysis confirmed significant positive associations among all variables (*p* < 0.001). Specifically, organizational climate was positively correlated with both professional identity (*r* = 0.681, *p* < 0.001) and job satisfaction (*r* = 0.462, *p* < 0.001). Job satisfaction was also positively correlated with professional identity (*r* = 0.506, *p* < 0.001).

**TABLE 3:** Descriptive statistics and correlation analysis of study variables (*N* = 580).

Variable	M (P25, P75)	1	2	3
1. Organizational climate	110.0 (97.0, 118.0)	1		
2. Professional identity	109.0 (90.0, 120.0)	0.681^∗∗∗^	1	
3. Job satisfaction	76.0 (68.0, 80.0)	0.462^∗∗∗^	0.506^∗∗∗^	1

^∗∗∗^ *p* < 0.001.

### 4.3. The Moderating Effect of Job Satisfaction

Hierarchical linear regression was conducted to assess the moderating effect of job satisfaction on the relationship between organizational climate and professional identity, controlling for significant demographic characteristics (annual shift type and monthly night‐shift frequency) (Table 4). All VIF values were below 2.5 and all tolerance values exceeded 0.4, indicating no serious multicollinearity. In Model 1, demographic variables were not significantly associated with professional identity (adjusted *R* ^2^ = 0.017). In Model 2, both organizational climate (*β* = 0.709, *p* < 0.001) and job satisfaction (*β* = 0.106, *p* < 0.001) were positively associated with professional identity (adjusted *R* ^2^ = 0.572). In the final Model 3, a significant interaction between organizational climate and job satisfaction (*β* = 0.086, *t* = 2.911, *p* < 0.01) was observed (adjusted *R* ^2^ = 0.578). These findings suggest that job satisfaction significantly moderates the association between organizational climate and professional identity.

**TABLE 4:** Moderating effect of job satisfaction on the relationship between organizational climate and professional identity (*N* = 580).

Variable	Model 1			Model 2			Model 3				
	*β*	*t*	*P*	*β*	*t*	*p*	*β*	*t*	*p*	VIF	Tolerance
Control variables											
Annual shift type	0.093	1.766	0.078	0.068	1.964	0.050	0.065	1.877	0.061	1.649	0.606
Monthly night shift frequency	−0.064	−1.206	0.288	0.000	−0.005	0.996	0.001	0.027	0.978	1.647	0.607
Main effects											
Organizational climate				0.709	24.693	< 0.001	0.673	21.707	< 0.001	1.320	0.758
Job satisfaction				0.106	3.676	< 0.001	0.126	4.276	< 0.001	1.194	0.838
Interaction effect											
Org. climate × job satisfaction							0.086	2.911	0.004	1.199	0.834
Model fit indices											
Adjusted *R* ^2^	0.017			0.572			0.578				
F value/*p*	5.924/0.003			194.688/< 0.001			159.468/< 0.001				
Δ*R* ^2^	0.020			0.575			0.581				

*Note:* Dependent variable: professional identity.

**FIGURE 1:**
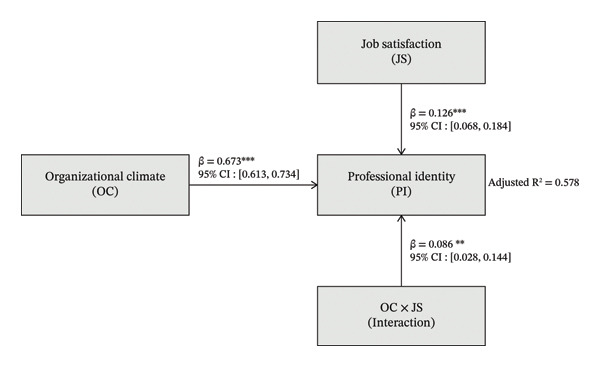
Research model of the moderating effect of job satisfaction on the relationship between organizational climate and professional identity. Note: ^∗∗∗^ *p* < 0.001, ^∗∗^ *p* < 0.01. OC = organizational climate; JS = job satisfaction; PI = professional identity. The final model explained 57.8% of the variance in professional identity (adjusted *R* ^2^ = 0.578).

As shown in Figure 1, organizational climate was positively associated with professional identity (*β* = 0.673, 95% CI [0.613, 0.734], *p* < 0.001). Job satisfaction was also positively associated with professional identity (*β* = 0.126, 95% CI [0.068, 0.184], *p* < 0.001). Importantly, the interaction term (organizational climate × job satisfaction) was significantly associated with professional identity (*β* = 0.086, 95% CI [0.028, 0.144], *p* = 0.004), indicating a positive moderating effect. The final model explained 57.8% of the variance in professional identity (adjusted *R* ^2^ = 0.578).

### 4.4. Simple Slopes Analysis of the Moderating Effect

A simple slopes analysis was conducted to examine the association between organizational climate and professional identity at three levels of job satisfaction: the mean, high (mean + 1 SD), and low (mean − 1 SD). As shown in Table 5, the positive association between organizational climate and professional identity remained statistically significant across all levels of job satisfaction (*p* < 0.001). However, the strength of this association increased as job satisfaction increased, with standardized coefficients rising from 0.64 (at low job satisfaction) to 0.76 (at high job satisfaction). This pattern confirms that higher job satisfaction significantly strengthens the positive association between organizational climate and professional identity.

**TABLE 5:** Simple slopes analysis: moderating effect of job satisfaction at different levels (*N* = 580).

Level of job satisfaction	*β*	*t*	*p*	95% CI (lower)	95% CI (upper)
Low level (−1 SD)	0.64	14.434	< 0.001	0.553	0.727
Average (mean)	0.70	21.707	< 0.001	0.636	0.763
High level (+1 SD)	0.76	24.789	< 0.001	0.699	0.819

Abbreviations: CI, confidence interval; SD, standard deviation.

## 5. Discussion

### 5.1. The Status of Nursing Professional Identity

Nurses in our study exhibited a moderate level of professional identity, with a median score of 109.0 (IQR: 90.0, 120.0). This score is higher than those reported in the earlier literature [19], suggesting improvement in the professional identity of the nursing workforce. This improvement may be attributed to the unique societal contributions made by nursing staff during the COVID‐19 pandemic [20]. Nurses played a critical role in patient care and infection management to support epidemic prevention and control, and their contributions have been widely recognized by society, which has reinforced their sense of professional worth and identity [20].

### 5.2. Impact of Shift Work and Mitigation Strategies

Comparison of professional identity scores across demographic and occupational groups showed that nurses on rotating shifts and those with frequent night shifts had significantly lower professional identity scores. Shift work and frequent night shifts not only disrupt circadian rhythms but can also induce emotional exhaustion, leading to burnout and a lower sense of professional identity [21]. Therefore, it is crucial to view nurses as “whole persons” whose physical and mental health are integral to their professional development. Measures should be taken to mitigate the negative impacts of the shift work system, including scientific scheduling optimization [22], strict protection of rest periods [23], and the provision of additional health support or compensation for night‐shift staff [24]. Meanwhile, hospital administrators should institutionalize comprehensive support systems, including psychological counseling, mindfulness training, and proactive career mentorship to foster professional well‐being [25]. These measures aim to help nurses maintain a professional identity even in high‐pressure environments [25].

### 5.3. The Relationship Between Organizational Climate and Professional Identity

This study confirmed a significant positive correlation between organizational climate and professional identity (*r* = 0.681, *p* < 0.001). This finding is consistent with the literature, which indicates that higher levels of perceived organizational support are associated with stronger professional identity [26]. Organizational climate is a sociopsychological construct that shapes a nurse’s dignity, autonomy, and sense of belonging within the emotional and cultural environment [27]. This finding aligns with the JD‐R model, in which a positive organizational climate functions as a beneficial job resource that leads to high work engagement and positive work performance [28]. When nurses perceive their work environment as supportive, it buffers against the physical and psychological stress associated with high job demands [29]. According to self‐determination theory, a positive organizational climate satisfies the intrinsic needs for competence and relatedness, thereby fostering a stronger professional identity [30].

These findings suggest that nursing managers should optimize job resources by creating a climate of psychological safety and empowerment to cultivate professional identity. This includes implementing shared decision‐making models that empower nurses to take control over their practice. By shifting from a top‐down management style to a transformational leadership style, managers can enhance nurses’ sense of autonomy and professional worth [31]. Additionally, developing social resources, such as mentorship programs and peer support networks, is also essential in buffering stress and improving professional identity [32]. By fostering a culture where nurses feel “seen” and “heard,” organizations can effectively reinforce shared values and strengthen professional identity [33].

### 5.4. The Moderating Role of Job Satisfaction

This study further reveals that job satisfaction positively moderates the positive association between organizational climate and professional identity. This implies that the association between supportive organizational climate and professional identity is stronger when nurses have higher job satisfaction [34]. According to Alderfer’s Existence, Relatedness, and Growth (ERG) Theory, employees possess three core levels of needs, and the fulfillment of lower‐level needs subsequently activates higher‐level needs. In this context, a positive organizational climate satisfies basic survival and relational needs, thereby fostering job satisfaction. When nurses feel valued, supported, and recognized, their increased satisfaction prompts them to proactively link organizational opportunities to their own career development [35]. This positive interpretation allows them to internalize organizational support into professional identity and commitment [35]. Conversely, if job satisfaction is low, emotional exhaustion or a perceived lack of value can erode professional self‐concept, hinder career planning, and ultimately trigger turnover intentions [36].

This moderating mechanism suggests that enhancing professional identity must address both objective environmental factors (e.g., organizational climate) and subjective emotional experience (e.g., job satisfaction). Therefore, nursing managers should take systematic interventions to enhance job satisfaction. They should focus on providing emotional support and professional supervision, reinforcing their sense of work meaning, optimizing clinician–patient communication, and establishing equitable incentive mechanisms. These measures are essential to maximizing the long‐term benefits of the organizational environment on professional identity.

### 5.5. Implications for Policy and Practice

Our study has significant implications for nursing practice in improving professional identity. First, hospitals should foster a supportive organizational climate and focus on resource availability and an empowering organizational culture. Second, hospitals should integrate monitoring and enhancing nurse job satisfaction into the routine management system, focusing on improving nurses’ perception of work meaning and professional growth. Third, administrators should simultaneously consider the dual impact of the objective environment (organizational climate) and subjective experience (job satisfaction) when designing interventions to improve professional identity. This holistic approach is essential to fostering a stable and internalized professional identity among nursing staff.

### 5.6. Limitations

Despite its insights, this study acknowledges several limitations. First, the cross‐sectional design precludes establishing causal relationships between organizational climate and professional identity. Future longitudinal research is needed to track temporal changes and facilitate causal inferences. Second, all data were collected via self‐reports, which may introduce social desirability bias. Future studies should incorporate objective indicators, such as retention rates and performance evaluations, to improve measurement accuracy. Third, the model did not account for potential confounders such as resilience or personality traits. Future studies should expand the scope of variables to get a more comprehensive understanding of the mechanisms driving professional identity.

## 6. Conclusion

The research verifies that there is a positive correlation between the organizational climate and nurses’ professional identity. Significantly, job satisfaction acts as a positive moderator in this relationship. Higher levels of job satisfaction reinforce the favorable impact of the organizational climate on professional identity. These results imply that interventions designed to enhance nurses’ professional identity should implement a dual‐strategy approach that optimizes the objective organizational climate while improving nurses’ subjective job satisfaction concurrently. Future longitudinal investigations are necessary to determine causality and explore additional moderating mechanisms.

## Author Contributions

Juan Lai: study design, data analysis, data interpretation, visualization, and writing an original draft. Chong Chen: data collection, data analysis, data interpretation, visualization, and review and editing. Huijun Liu: data interpretation, visualization, validation, and review and editing.

## Funding

No funding was received for this secondary analysis.

## Disclosure

All authors read and approved the final manuscript.

## Ethics Statement

This study was conducted in accordance with the Declaration of Helsinki, and ethical approval was granted by the Ethics Committee of Xiangya Hospital, Central South University (Approval No. 202312254). All participants provided informed consent at the time of participation and were informed about and consented to the analysis of survey results.

## Consent

The authors have nothing to report.

## Conflicts of Interest

The authors declare no conflicts of interest.

## Data Availability

The full dataset for this study is not publicly available due to data protection protocols at Xiangya Hospital, Central South University; however, deidentified, summary‐level data can be shared upon reasonable request to the corresponding author.
